# Towards a West of England Medical Journal

**Published:** 1989-05

**Authors:** 


### TOWARDS A WEST OF ENGLAND MEDICAL JOURNAL

This is the last number of the *Bristol Medico-Chirurgical Journal* which will be sent gratis to all doctors in the West of England, as has been our practice during the past 3 years. The commercial sponsorship which made this possible has now ended. In future it will be distributed to members of the Bristol Medico-Chirurgical Society and to independent subscribers.

We are fortunate that the Nuffield organisation has agreed to sponsor the journal for a period and our immediate future is again secure.

**We invite West of England doctors who wish to continue to receive the journal to complete and return the order form enclosed herewith.**

During these past three years this journal has in effect been a *West of England Medical Journal*. All doctors in the region have received it, many of them have contributed to it. It has printed abstracts of the papers given at many of the West of England specialist clubs. The Bristol Medical Chirurgical Society now feels that the time has come for the journal to become a true West of England journal with an extended constituency. It has invited all the specialist clubs and local medical societies in the West of England to join with them in an association to produce a *West of England Medical Journal* to which they would all contribute and which would be received by all their members. Such a joint venture could produce a really good journal which would become a force in British medical journalism. There is surely a need for other quality general medical journals in Britain besides the BMJ and the Lancet. The recently formed *Scottish Medical Journal* is one such. A *West of England Medical Journal* could be another with an additional emphasis on west country affairs and history.

The initial response is very positive. A meeting to discuss the project will be called in the early summer and we hope to be able to report progress in our next number.

